# The Reform of Medical Education in France

**Published:** 1911-01-28

**Authors:** 


### SPECIAL ARTICLES.

### THE REFORM OF MEDICAL EDUCATION IN FRANCE.

For some years past it has been recognised by those competent to judge that all is not well with medical education in France; but the fact that no steps have been taken to improve the methods of education now in vogue is due, not to apathy on the part of would-be reformers, but to the impracticability and lack of precision of the reforms hitherto advocated. Quite recently a movement was started, which resulted in the calling together of a Congress of Practitioners, before which the question of medical education was carefully thrashed out, and a number of resolutions and plans of reform discussed. Much opposition to these was encountered, not only from the lay authorities, but also from members of the medical profession, and up to the present no steps have been taken to put any of the suggestions into practice. This has led Dr. Leredde,* an ardent reformer, to investigate the whole matter in its various aspects. Much of the opposition which the reformers have encountered from members of the medical profession comes from imperfectly understanding the points under dispute.

### The Position in France.

The conditions under which medical education is carried out in France are widely different from those with us. Every medical school in that country is under a central authority, which dictates rules and regulations, conditions of attendance, and admission to examinations. In other words, no school has autonomy of any sort, or can be styled in any way a private school, as are the majority of our schools. Professors are bound to teach on stereotyped lines in order to fulfil the conditions under which they hold their appointments. Consequently teaching has not advanced along with changes in medical knowledge, and as a result the technical side of medicine has been neglected in favour of the theoretical. The main point in the plans of the reformers is the modification of this excessive centralisation. It is a curious fact that for most Frenchmen the word school signifies merely a collection of lecture-rooms. Consequently they imagine that a medical school can be conducted in the same way as a primary school.

### What is a Medical School?

In the opinion of the reformers a medical school, whether large or small, is an association, not of lecture-rooms, but of workshops (*ateliers*). Technical teaching must therefore be substituted for that in vogue at present, which is neither technical, practical, clinical, nor scientific in the true sense of these words, and is not even properly organised on theoretical lines. All teachers in sympathy with the reform movement believe that a school must be adapted to the needs of its pupils, and consequently demand the abolition of this centralisation. They recognise that they themselves have learnt medicine in workshops, not in lecture-rooms. They have been taught what they know by working in *collaboration* with their teachers, and have, to a large extent, escaped the restrictions, regulations, programmes, and even, in some cases, the examinations of the administrative authority. "Have we or have we not," demands the professor, "the right of asking that our pupils shall be instructed in the same way as their masters? If these last have received a technical training, by what right can we refuse the same to the former? If masters have been trained in medical workshops, why should not pupils be?"

### A Medical Workshop.

No answer to these questions has as yet been given by the opponents of the scheme, and while awaiting a reply Leredde sketches out what he means by a medical workshop. Three conditions are necessary for technical teaching: (1) The student must spend all his time at the hospital and in the laboratory. The latter-day student, however, bound as he is by rules and regulations, lives in lecture-rooms and libraries at such times as he is unable to prepare for his examinations at home. (2) There must be collaboration between teacher and pupil. They must be associated in a common task. The teacher must not be a professor delivering a course of lectures, but a man observing and gaining experience. The pupil must not be a student listening and taking notes, but an apprentice exercising his powers of observation and gaining experience by contact with his teacher. It is quite possible to imagine a state of things in which teaching is entirely freed from theory. This last can be got from the books; but it is unthinkable that medical instruction should exist without co-operation between master and pupils. In fact, in every official school, though hidden away out of sight, a technical department exists, for no school could continue without it. As the result of co-operation of master and pupils there is formed around the former a small family, as it were, and in this way technical teaching presupposes, authorises, and determines mutual education. (3) The pupil must have free choice of a master, and must himself determine the clinic and the laboratory in which he will work. Rules may have to be enforced to limit the number of pupils received into any one class; for the hospital was founded for patients, not for students, and even medical teaching must be subordinated to the material and moral welfare of the sick. But on no pretext whatever should regulations be framed obliging pupils to join clinics they have not chosen of their own accord; for the master that suits A may be uncongenial to B, and technical teaching exacts a moral lien between master and pupil.

This idea of education by collaboration or co-operation is not understood by the Central Administration of Public Instruction, since the one aim and object of this body is to set up dogmatic instruction by *affirmation*. With regard to the teaching of theory, it should be borne in mind that technical instruction is the main matter, theory but its complement. The question of education in science is a more difficult one. The present administration has for a long time introduced trouble into the schools on the plea of developing scientific instruction. Medical opinion has protested in vain against this innovation. The authorities would seem to have acted on the supposition that students were made for the laboratories and not laboratories for the students, much in the same way as the military authorities consider soldiers made for barracks. The reformers would do away with laboratories as such, and make them an appendage of the hospital. In other words, scientific instruction would become technical. It is true that the laboratories at present attached to the hospitals are in many cases imperfect; but then this imperfection comes from the very nature of the instruction given in the hospitals. When the hospitals become the teaching centres the laboratories will, *ipso facto*, grow in importance and efficiency. The preliminary sciences should be taught in central institutes, and if the chairs of theoretical teaching are suppressed, as would seem desirable, the professors at present occupying them should be transferred and given charge of these institutes.

### The Question of Examinations.

In addition to the reforms outlined above, those who are in sympathy with the idea of technical medical education would like to see radical changes introduced into the system of examination for medical degrees. Examinations in a technical school must be themselves of a technical nature. Consequently the present type of examination must be abolished, and no student allowed to work directly for an examination. This final test must be used by the examiners so as to acquire a knowledge of the training a student has received, and not, as at present, to prove his capacity for work and cramming. The candidate will, of course, have to produce evidence of having done regular work in the medical workshop, and it will be necessary to insist on a certain minimum of time being devoted to each of the subjects included in the curriculum.

### Conclusions.

The author brings his pamphlet to a close by the consideration of certain general propositions into which it is not necessary to follow him here. He agrees, however, that there are certain obstacles to the organisation of medical teaching in France which are perhaps more difficult to surmount in that than in any other country, since every French school, from the primary to those of the various Faculties, is considered a sort of clock dependent on external administration and compelled to obey the decrees of this last. The interests of the bureaux are in direct opposition to the autonomy of the schools. The medical fraternity has been among the first to understand the disadvantages of a bad system of teaching, and it is hoped that the public in its turn will commence to comprehend the evil. Hitherto conflicting political interests have obscured the education problem. Whether professional or otherwise, a school is a centre of education and not merely a place where things are taught. That essential of education, the personal effort of the pupil, cannot be created by programmes and rules, in fact these last act in most cases as a direct obstacle. The essential of education depends on the authority and activity of the master, on his direct action on the pupil, and the degree of intimacy which exists between the two. It is an unfortunate mistake to submit the teacher to texts and rules upon which he has had no opportunity of reflecting, and which do not proceed from his own brain or from that of the teaching body to which he belongs. Every school is a family, with the master as its head.

* Le Programme des Réformes de l'enseignement Médical. L'Ecole de Médecine Technique. Par M. le Dr. Leredde. Extrait de *La Tribune Médicale*, de 8 et 15 Octobre 1910. (Paris : Imprimerie de Vaugirard, 1910.)

